# Influence of Diabetes Mellitus on the Development of Multidrug‐Resistant Tuberculosis (MDR‐TB) in Relation With Genetic Mutations of *M. tuberculosis* Isolates in Ethiopia

**DOI:** 10.1155/cjid/7830270

**Published:** 2026-05-30

**Authors:** Aynias Seid, Semira Nureddin

**Affiliations:** ^1^ Department of Biology, College of Natural and Computational Sciences, Debre-Tabor University, Debre-Tabor, Ethiopia; ^2^ Department of Biology, College of Natural and Computational Sciences, Woldia University, Woldia, Ethiopia, wldu.edu.et

**Keywords:** comorbidity, diabetes mellitus, drug resistance, mutations, tuberculosis

## Abstract

In nations with developing healthcare infrastructures, the dual burden of Type 2 diabetes mellitus (T2DM) and tuberculosis represents a critical health challenge. The aim of this study is to investigate the influence of T2DM on the development of multidrug‐resistance tuberculosis (MDR‐TB) in relation to genetic mutations of *Mycobacterium tuberculosis* in Ethiopia. A facility‐based cross‐sectional study was carried out between 2023 and 2024. Genetic mutations associated with drug resistance in both diabetic and nondiabetic groups were detected using line probe assays (LPAs). Diagnosis of T2DM was performed using fasting blood glucose and glycated hemoglobin levels. Data were analyzed with SPSS software, and logistic regression models were performed. Our findings revealed that 44 of the 182 participants (24.2%) were TB–T2DM, whereas 138 (75.8%) were TB with nondiabetic groups. Multivariate analysis displayed that the prior TB treatment history (aOR: 2.51, 95%CI: 1.02–6.19, *p* = 0.046) and a family history of T2DM (aOR: 2.25, 95% CI: 1.10–4.60, *p* = 0.031) were independently associated with an increased risk of TB–T2DM comorbidity. Genetic analysis identified a total of 50 mutations (22 *rpoB*, 22 *katG*, and 6 *inhA* promoter) associated with resistance to RIF and INH drugs among 31 drug‐resistant *M. tuberculosis* isolates, which were significantly more common in TB–T2DM comorbid patients (*p* = 0.009). The *rpoB* S450L and *katG* S315T1 were the predominant mutations typically associated with high‐level RIF and INH resistance, respectively, and high biological fitness, allowing for successful transmission within the community. Among the dual mutations identified in MDR‐TB isolates, only the *rpoB* UN + *katG* S315T1 mutation was significantly more prevalent in nondiabetics (*p* = 0.04) compared to the TB–T2DM comorbid group. Multivariate analysis revealed that prior TB treatment history (aOR: 4.00, 95% CI: 1.31–12.50, *p* = 0.014) and TB–T2DM comorbidity (aOR: 3.12, 95% CI: 1.02–9.10, *p* = 0.042) were significantly associated with an increased likelihood of MDR‐TB. This increased odd of resistance in comorbid patients highlights the need for bidirectional T2DM screening for all TB patients, particularly those with a history of prior TB treatment or a family history of diabetes. A genome‐wide association study into the mutation patterns responsible for DR is crucial to inform the development of targeted, effective strategies moving forward.

## 1. Introduction

Diabetes mellitus (DM) is a group of noncommunicable chronic metabolic disorders characterized and identified by the presence of hyperglycemia with disturbances of carbohydrate, fat, and protein metabolism. Type 1 DM (T1DM) is an autoimmune disease where the antibodies attack and destroy insulin‐producing ß‐cells of islets of Langerhans, making patients insulin‐dependent or requiring lifelong insulin therapy [1–4]. In contrast, the more prevalent diabetes subtype is Type 2 DM (T2DM), a condition that results from a combination of multigene predisposition and environmental triggers [4, 5], where the body cell either does not produce enough insulin or becomes resistant to it, thereby resulting in hyperglycemia and making the insulin ineffective over time [2, 3]. Tuberculosis (TB), on the other hand, is a communicable infectious disease caused by a single *Mycobacterium tuberculosis* complex strain. There is a bidirectional relationship between DM and TB with epidemiological implications [6], as T2DM is becoming more prevalent in TB‐endemic settings [7]. Worldwide, the incidence of TB is highest among individuals with compromised immune systems, HIV infection, or diabetes. Currently, Africa is facing increasing rates of DM and TB, presenting significant public health challenges. The burden of co‐morbid TB–T2DM is estimated to be higher than TB–HIV co‐infection, particularly in low‐ and middle‐income countries (LMICs) like Ethiopia [8, 9].

The co‐occurrence of TB and DM diseases can be aggravated by acquisition of genetic mutations conferring anti‐TB drug resistance (DR) in high‐burden settings [10, 11]. During comorbidity, DM has a negative impact on TB treatment outcomes with the risk of more likely developing DR‐TB disease and can reduce or alter the phagocytes’ immune function and aggravate the condition of TB patients compared to those TB patients without DM [12], while TB can also worsen glucose metabolism disorders and make it difficult to control blood sugar levels in patients with DM [10]. Factors associated with the comorbidity of DM and TB include age, sex, presence of TB, hypertension, family history of DM, alcohol consumption, and place of residence [13, 14].

The prevalence of TB patients with DM comorbidity is higher than TB patients without diabetes [15, 16], which gives a great recommendation on bidirectional screening of the two diseases and also limited evidence on the effectiveness of specific TB testing in individuals with DM and specific DM tests for patients with TB [17, 18].

The management of TB–T2DM comorbidity is different from managing either disease alone. Findings revealed that individuals with the co‐occurrence of TB–T2DM are more likely to test positive on smears or cultures for pulmonary TB (PTB) and have higher bacillary loads [7, 19]. This comorbidity leads to lower plasma levels of anti‐TB drugs [20] and changes in the immune system [9, 21] and a higher likelihood of developing multidrug‐resistant tuberculosis (MDR‐TB) [13, 14, 18, 19], all of which adversely affect the effect of drug treatment [7] and lead to a significant increase in recurrence rate or risk of treatment failure and mortality in TB patients [19]. MDR‐TB is a condition in which *M. tuberculosis* is at least resistant to the key first‐line drugs rifampin (RIF) and isoniazid (INH) in anti‐TB treatment.

Like HIV–TB co‐infection [22], the DM‐TB comorbidity has also been described as a determinant in the development of DR in relation to genetic mutations in *M. tuberculosis*. The most frequent RIF resistance‐conferring *rpoB* mutations, encoding the *β* subunit of RNA polymerase, are due to amino acid changes at codons 450, followed by 445, 435, and 430 [23, 24]. Mutations in the *katG* at codon 315 and the promoter region of the *inhA* genes are the major mechanisms of high‐level and low‐level INH resistance. Genome sequencing has to improve our understanding of resistance mechanisms [25]. Currently, the molecular detection of genetic mutations associated with DR can aid in the development of a predictive diagnosis of DR in TB. A recent report has indicated that *M. tuberculosis* strains resistant to RIF and isolated from TB patients co‐infected with HIV carry more *rpoB* mutations that confer resistance, and these strains are linked to decreased fitness in the absence of the drugs [23]. Although several findings show that TB–DM comorbidity results in treatment failure, extended sputum positivity, risk of developing DR‐TB, and higher mortality rates, the relationship between the TB–T2DM comorbidity and MDR‐TB, particularly in relation to genetic variations within *M. tuberculosis* in Ethiopia, remains poorly understood. The study aimed to evaluate the influence of T2DM on the development of MDR with genetic mutations in *M. tuberculosis*.

## 2. Materials and Methods

### 2.1. Study Design, Settings, and Period

A facility‐based cross‐sectional study was conducted between 2023 and 2024 at selected three hospitals in the Amhara Regional State of Ethiopia. According to the latest 2025 WHO Global TB report [12], Ethiopia has an estimated annual TB incidence rate of roughly 146–188 per 100,000 populations and Amhara Region remains one of the high‐burden areas for TB in the country. A total of 512 both newly diagnosed and previously treated (retreatment) TB patients visited the University of Gondar Comprehensive Specialized Hospital (UoGCSH), Debre‐Tabor Comprehensive Specialized Hospital (DTCSH), Felege‐Hiwot Specialized Hospital (FHSH), which serve as the primary referral hubs for MDR‐TB and complex TB cases in the Central Gondar, South Gondar, and West Gojam Amhara Zones. Out of which, 182 GeneXpert‐positive PTB patients were screened who met the strict inclusion criteria for molecular analysis during the study period. A consecutive sampling technique was employed to recruit study participants, who are with age above 18 years, culture‐positive *M. tuberculosis* isolates, and willingness to provide sign written informed consent and a face‐to‐face interview. Patients with extra‐pulmonary TB, culture‐negative results, or those diagnosed with nontuberculosis mycobacteria (NTM) were excluded from the study. Study participants were divided into the TB–T2DM comorbid and only TB‐non T2DM groups.

### 2.2. Laboratory Procedures

#### 2.2.1. Diagnosis of PTB

All participants were given instructions on how to properly collect a high‐quality sample and prevent contamination with saliva in a well‐ventilated area specifically designated for this purpose. All patients suspected of having TB who visited the selected facilities during the study period were given a 4 mL sputum sample “on the spot” for clinical diagnosis of PTB using GeneXpert® MTB/RIF assay in accordance with the manufacturer’s instructions [26]. Following this, a single 5–10 mL “morning” sputum sample was collected from each GeneXpert‐confirmed PTB patient by trained laboratory technologists at each facility using a properly labeled disposable Falcon tube. All of the collected sputum samples were kept in a cold chain and transported to the UoGCSH TB culture laboratory for laboratory analysis.

#### 2.2.2. Diagnosis of DM

Venous blood (3–4 mL) was collected in EDTA (ethylenediamine‐tetra‐acetic acid) tubes for all study participants in the morning at each hospital. The collected biological samples were transported on ice. The samples were then dispensed into serum separator gel tubes and centrifuged at 3000 rpm for 5 min to isolate leukocytes and produce plasma. Two milliliters of the plasma were used to measure fasting blood glucose (FBG) levels and hemoglobin A1c (HbA1c) levels within 4–6 h after sample collection. T2DM was diagnosed based on WHO criteria: FBG levels ≥ 126 mg/dL and/or glycated hemoglobin (HbA1c) levels ≥ 6.5 [3]. To ensure accuracy and differentiate from transient stress hyperglycemia, HbA1c was used as the primary longitudinal marker of glycemic control.

#### 2.2.3. Sample Preprocessing, Culturing, and Identification of *M. tuberculosis* Isolates

Sputum samples were decontaminated using an equal volume of N‐acetyl‐L‐cysteine/sodium hydroxide (NALC/NaOH) with a final concentration of 2% NaOH [27]. The processed sediment was inoculated (10 L) into Löwenstein–Jensen (LJ) media and incubated at 37°C for up to 8 weeks, with readings taken weekly and recorded in the lab logbook. The *M. tuberculosis* complex (MTBC) was confirmed via Ziehl–Neelsen (ZN) staining [28] and the SD BIOLINE TB Ag MPT64 Rapid® test (Standard Diagnostics, Inc., South Korea) to differentiate from NTM [29].

#### 2.2.4. Molecular Analysis of *M. tuberculosis* Clinical Isolates

Genomic DNA was extracted from culture‐positive *M. tuberculosis* isolates using the GenoLyse® DNA extraction kit [30, 31]. Molecular characterization of mutations in the *rpoB* (RIF resistance), *katG* (high‐level INH resistance), and *inhA* promoter (low‐level INH resistance) genes was performed using GenoType® MTBDRplus v.2.0 line probe assay (LPA) (Hain LifeScience, Germany) at the UoGCSH TB culture laboratory according to the manufacturer’s protocol [30, 32]. LPA was performed on cultures, not direct sputum. The LPA experiment was conducted by following the steps of DNA‐kit strip technology. All laboratory experiments were conducted following standard operating procedures (SOPs). For the LPA of the test, DNA from the *M. tuberculosis* H37Rv strain (ATCC 27294) and molecular‐grade water were used as positive and negative controls, respectively.

### 2.3. Data Collection and Statistical Analysis

The relevant sociodemographic, clinical data, and behavioral information of the study patients were collected through face‐to‐face interviews using semistructured questionnaires. Data were collected by well‐trained health workers. Data were entered and analyzed using SPSS Version 26.0 software. Categorical variables were compared using Pearson’s chi‐square (*χ* ^2^) or Fisher’s exact test used for cells with small expected frequencies. Bivariate and multivariate logistic regression models were used to calculate odds ratios (OR) with 95% confidence intervals (CIs) to identify independent predictors of MDR‐TB and TB–T2DM comorbidity. Statistical significance was set at *P* value less than 0.05.

## 3. Results

### 3.1. Sociodemographic and Clinical Characteristics of Participants

Among the 182 participants, 44 (24.2%) were diagnosed with TB–T2DM co‐morbidity, and the remaining 138 (75.8%) had TB without T2DM, as shown in Table 1. Regarding sociodemographic factors, the age distribution was examined with a notable difference between the two groups. Older age (age ≥ 45 years) was more frequent in the TB–T2DM comorbid group (40.9%) compared to the TB‐only group (26.1%), though this did not reach significance (*χ* ^2^ = 0.048,  *p* = 0.061). Similarly, other sociodemographic factors such as sex, residency, and household size did not differ significantly between the two groups (*p* > 0.05).

**TABLE 1:** Sociodemographic and clinical characteristics of study participants based on TB–T2DM co‐morbidity status (*n* = 182).

Variables	Categories	TB–T2DM (*n* (%))	TB without T2DM (*n* (%))	Total (*n* (%))	*χ* ^2^/Fisher’s	*p* value
Sex	Male	27 (61.4)	84 (60.9)	111 (61.0)	0.550	0.953
	Female	17 (38.6)	54 (39.1)	71 (39.0)		

Age group	≥ 45	18 (40.9)	36 (26.1)	54 (29.7)	0.048	0.061
	18–44	26 (59.1)	102 (73.9)	128 (70.3)		

Residency	Rural	20 (45.5)	69 (50.0)	89 (48.9)	0.363	0.599
	Urban	24 (54.5)	69 (50.0)	93 (51.1)		

Number of households	1–3	22 (50.0)	52 (37.7)	74 (40.7)	0.102	0.147
	≥ 4	22 (50.0)	86 (62.3)	108 (59.3)		

Prior TB treatment history	Pretreated	12 (27.3)	17 (12.3)	29 (15.9)	0.020	0.018^∗^
	New cases	32 (72.7)	121 (87.7)	154 (84.1)		

Window opening practice	Yes	17 (38.6)	52 (37.7)	69 (37.9)	0.523	0.909
	No	27 (61.4)	86 (62.3)	113 (62.1)		

Previous contact with TB	Yes	19 (43.2)	36 (26.1)	55 (30.2)	0.027	0.032^∗^
	No	25 (56.8)	102 (73.9)	127 (69.8)		

History of alcohol drinking	Yes	10 (22.7)	31 (22.5)	41 (22.5)	0.560	0.971
	No	34 (77.3)	107 (77.5)	141 (77.5)		

History of cigarette smoking	Yes	4 (9.1)	11 (8.0)	15 (8.2)	0.513	0.814
	No	40 (90.9)	127 (92.0)	167 (91.8)		

	Total (n/%)	44 (24.2)	138 (75.8)	182 (100)		

*Note:* *χ* ^2^, Pearson’s chi‐square test; T2DM, Type 2 diabetes mellitus.

Abbreviations: TB, tuberculosis.

^∗^Statistically significant.

Notably, pretreated TB patients were significantly more prevalent in the comorbid group (27.3%) than in the TB‐only group (12.3%), with a statistically significant difference (*χ* ^2^ = 0.020, *p* = 0.018). Additionally, a history of contact with TB was significantly associated with the TB–T2DM comorbid group (43.2% vs. 26.1%, *p* = 0.032). Behavioral factors, including window opening habit and alcohol and tobacco use, were uniformly distributed across both groups (*p*  > 0.05) (Table 1).

### 3.2. Patient‐Specific Risk Factors Associated With T2DM–TB Comorbidity

The study assessed 182 participants to identify the significant sociodemographic, clinical, and behavioral factors linked to TB–T2DM co‐occurrence as shown in Table 2. In the bivariate analysis, a family history of T2DM was significantly associated with increased odds of TB–T2DM comorbidity (cOR: 2.47, 95% CI: 1.11–5.50, *p* = 0.027). After adjusting for cofounders, it was also independently associated with an increased risk of comorbidity (aOR: 2.25, 95% CI: 1.10–4.60, *p* = 0.031). Similarly, multivariate logistic regression identified prior TB treatment history as a significant predictor for TB–T2DM comorbidity (aOR: 2.51, 95% CI: 1.02–6.19, *p* = 0.046).

**TABLE 2:** Logistic analysis of risk factors associated with TB–T2DM comorbidity (*N* = 182).

Variables	Categories	TB–T2DM (*n* (%))	TB without T2DM (*n* (%))	Bivariate		Multivariate	
				cOR (95% CI)	*p* value	aOR (95% CI)	*p* value
Sex	Male	27 (61.4)	84 (60.9)	1.02 (0.51–2.04)	0.95	—	—
	Female	17 (38.6)	54 (39.1)	1 (reference)			

Age group	≥ 45	18 (40.9)	36 (26.1)	1.96 (0.96–4.00)	0.06	1.78 (0.85–3.70)	0.128
	18–44	26 (59.1)	102 (73.9)	1 (reference)		1 (reference)	

Residency	Rural	20 (45.5)	69 (50.0)	0.83 (0.42–1.65)	0.60	—	—
	Urban	24 (54.5)	69 (50.0)	1 (reference)			

Prior TB treatment history	Pretreated	12 (27.3)	17 (12.3)	2.67 (1.16–6.15)	0.02^∗^	2.51 (1.02–6.19)	0.046^**∗**^
	New cases	32 (72.7)	121 (87.7)	1 (reference)		1 (reference)	

Previous contact with TB	Yes	19 (43.2)	36 (26.1)	2.15 (1.06–4.37)	0.034	1.89 (0.90–3.96)	0.094
	No	25 (56.8)	102 (73.9)	1 (reference)		1 (reference)	

Family history for T2DM	Yes	13 (29.5)	20 (14.5)	2.47 (1.11–5.50)	0.027^∗^	2.25 (1.10–4.60)	0.031^**∗**^
	No	31 (70.5)	118 (85.5)	1 (reference)		1 (reference)	

Window opening practice	Yes	17 (38.6)	52 (37.7)	1.04 (0.52–2.09)	0.91	—	—
	No	27 (61.4)	86 (62.3)	1 (reference)			

Alcohol intake	Yes	10 (22.7)	31 (22.5)	1.02 (0.45–2.28)	0.97	—	—
	No	34 (77.3)	107 (77.5)	1 (reference)			

Cigarette smoking	Yes	4 (9.1)	11 (8.0)	1.15 (0.35–3.80)	0.81	—	—
	No	40 (90.9)	127 (92.0)	1 (reference)			

*Note:* NH, isoniazid; MDR, multidrug‐resistant; RIF, rifampicin; and TB–T2DM, tuberculosis with Type 2 diabetes mellitus.

Abbreviations: aOR, adjusted odds ratio; cOR, crude odds ratio; CI, confidence interval.

^∗^Statistically significant.

However, previous contact history showed association in bivariate analysis (cOR: 2.15, 95% CI: 1.06–4.37, *p* = 0.034), it was later not an independent predictor in the adjusted model (aOR: 1.89, 95% CI: 0.90–3.96, *p* = 0.094). Furthermore, other tested sociodemographic and behavioral factors, including age, sex, residency, window opening habit, and alcohol and tobacco use, were not significantly associated with the development of TB–T2DM comorbidity (*p* > 0.05) (Table 2).

### 3.3. Frequency of Genetic Mutations and DR Profiles

Among the 182 culture‐confirmed *M. tuberculosis* clinical isolates examined for resistance to RIF and INH drugs, a total of 50 genetic mutations were identified across the *rpoB, katG,* and *inhA* target genes. Of these, 22 *rpoB*, 22 *katG*, and 6 *inhA* promoter mutations associated with resistance to RIF, high‐level and low‐level INH, respectively, were recognized. The most frequent RIF‐resistance–associated mutation was *rpoB* S450L, found in seven individuals with TB–T2DM2 and three individuals with only TB cases, followed by *rpoB* UN (unknown mutations) detected in one individual with the T2DM–TB group and five individuals with the TB group. The most common INH‐resistance mutation was *katG* S315T1, which was observed in five isolates from individuals with the DM2–TB group and 13 isolates from TB‐only patients without T2DM.

Regarding the distribution of resistance profiles, patients with TB–T2DM comorbidity exhibited a significantly lower proportion of pan‐susceptible isolates (32 cases, 72.73%) compared to those nondiabetic TB‐only patients (119 cases, 86.23%), with a statistical significance (*p* = 0.028). Drug‐resistant isolates were significantly more prevalent in the TB–T2DM comorbid patients (12 cases, 27.27%) than in the TB‐only non‐diabetic group (19 cases, 13.77%), with a statistically significant difference (*p* = 0.009). All RIF mono‐resistance isolates were exclusively observed in TB‐only patients with nondiabetics (three cases, 2.17%) by mutation of *rpoB* H445Y in two and *rpoB* S450L in one case, while no RIF mono‐resistant isolate was detected among patients with TB–T2DM, without statistically significant (*p* = 1.0). INH mono‐resistance was more prevalent in TB with the nondiabetic group (seven cases, 5.07%), primarily associated with the most common *katG* S315T1 mutation conferring high‐level resistance in six cases and the *inhA* C‐15T mutation conferring low‐level resistance in one case, compared to those in TB–T2DM groups (two cases, 4.54%) with both linked to the *katG* S315T1 mutation, with no statistically significant (*p* = 0.78) (Table 3).

**TABLE 3:** Distribution of drug resistance profiles and frequency of genetic mutations based on TB–T2DM co‐morbidity (*N* = 182).

Variables/Categories	TB–T2DM co‐morbidity status in frequency and proportion (*N*, %)			
	TB–T2DM (*n* (%))	TB with non‐T2DM (*n* (%))	Total (*n* (%))	*p* value
Pan‐susceptible (INH/RIF) isolates	32 (72.73)	119 (86.23)	151 (82.97)	0.028**^∗^**
Any drug‐resistant isolates	12 (27.27)	19 (13.77)	31 (17.03)	0.009**^∗^**
Mono‐resistance				
RIF mono‐resistant isolates	0 (0.0)	3 (2.17)	3 (1.65)	
*rpoB* S450L	0 (0.0)	1 (0.72)	1 (0.55)	
*rpoB* H445Y	0 (0.0)	2 (1.45)	2 (1.10)	
INH mono‐resistant isolates	2 (4.54)	7 (5.07)	9 (4.95)	
*katG* S315T1	2 (4.54)	6 (4.35)	8 (4.40)	
*inhA* C‐15T	0 (0.0)	1 (0.72)	1 (0.55)	
MDR‐TB (both RIF and INH)	10 (22.73)	9 (6.52)	19 (10.44)	0.005**^∗^**
*rpoB* S450L + *katG* S315T1	2 (4.54)	0 (0.0)	2 (1.10)	0.055
*rpoB* S450L + *inhA* C‐15T	1 (2.27)	2 (1.45)	3 (1.65)	0.55
*rpoB* S450L + *katG* UN	4 (9.09)	0 (0.0)	4 (2.20)	0.055
*rpoB* H445Y + *katG* S315T1	0 (0.0)	2 (1.45)	2 (1.10)	0.15
*rpoB* UN + *katG* S315T1	1 (2.27)	5 (3.62)	6 (3.30)	0.04**^∗^**
*rpoB* D435V + *inhA* C‐15T	2 (4.54)	0 (0.0)	2 (1.10)	0.055
Total	44 (24.18)	138 (75.82)	182 (100)	

Abbreviations: INH, isoniazid; MDR‐TB, multidrug‐resistant tuberculosis; RIF, rifampicin; TB–T2DM, tuberculosis with Type 2 diabetes mellitus; UN, unknown mutation (no hybridization with wild‐type or mutation probes).

^∗^Statistically significant.

MDR‐TB prevalence was substantially higher in the TB–T2DM group (10 cases, 22.73%) compared to the nondiabetic group (nine cases, 6.52%) with statistically highly significant (*p* = 0.005). The distribution of mutation patterns exhibited variation between the TB–T2DM and nondiabetic groups, indicating distinct resistance mechanisms in this population. The dual mutations of the *rpoB* S450L + *katG* UN were prominent in the TB–T2DM comorbid group (four cases, 9.09%), followed by the combination of *rpoB* S450L + *katG* S315T1 and *rpoB* D435V + *inhA* C‐15T (two cases each, 4.54%) (*p*  >  0.05). Interestingly, the combination of *rpoB* UN + *katG* S315T1 mutation was significantly more frequent in TB with nondiabetic patients (five cases, 3.62%) compared to the TB–T2DM comorbid group (one case, 2.27%) (*p* = 0.04) as shown in Table 3. However, the infrequent prevalence of specific mutations in the study prevented the investigation of possible associations between mutation frequency and the development of TB–T2DM comorbidity.

### 3.4. Risk Factors Associated With the Development of MDR‐TB

As shown in Table 4, both bivariate and multivariate analyses highlight the significant factors linked with the risk of developing MDR‐TB. When examining the potent risk factors, bivariate analysis revealed that patients with an older age (≥ 45 years) were over 8 times more likely to develop MDR‐TB (cOR: 8.61, 95% CI: 2.92–25.40, *p* < 0.001) compared to the younger age (18–44) group. After adjusting for confounders, this association was continued in the multivariate analysis, with a highly significant risk of developing MDR‐TB (aOR: 6.33, 95% CI: 2.03–19.77, *p* = 0.001). The multivariate logistic analysis revealed that patients with a history of prior TB treatment were 4 times more likely to develop MDR‐TB (aOR: 4.00, 95% CI: 1.31–12.50, *p* = 0.014) compared to new cases. Additionally, the presence of TB–T2DM comorbidity was significantly associated with a threefold increase in the risk of developing MDR‐TB (aOR: 3.12, 95% CI: 1.02–9.10, *p* = 0.042).

**TABLE 4:** Logistic regression analysis between the significant factors associated with the development of MDR‐TB (*N* = 182).

Variables	Categories	MDR‐TB (n (%))		Total (n (%))	Bivariate		Multivariate	
		Yes	No		cOR (95% CI)	*p* value	aOR (95% CI)	*p* value
Sex	Male	9 (47.37)	102 (63.58)	111 (61.00)	1.86 (0.72–4.83)	0.21	—	—
	Female	10 (52.63)	61 (37.42)	71 (39.00)	1 (reference)			

Age group	≥ 45	14 (73.68)	40 (24.54)	54 (29.67)	8.61 (2.92–25.40)	< 0.001**^∗^**	6.33 (2.03–19.77)	0.001**^∗^**
	18–44	5 (29.32)	123 (75.46)	128 (70.33)	1 (reference)		1 (reference)	

Prior TB treatment history	Pretreated	9 (47.37)	20 (12.27)	29 (15.93)	6.43 (2.33–17.75)	< 0.001**^∗^**	4.00 (1.31–12.50)	0.014**^∗^**
	New cases	10 (52.63)	143 (87.73)	153 (84.07)	1 (reference)		1 (reference)	

History of previous TB contact	Yes	8 (42.11)	47 (28.83)	55 (30.22)	1.79 (0.68–4.74)	0.24	—	—
	No	11 (57.89)	116 (71.17)	127 (69.78)	1 (reference)			

Presence of TB–T2DM comorbidity	Yes	10 (52.63)	34 (20.86)	44 (24.18)	4.22 (1.59–11.19)	0.004**^∗^**	3.12 (1.02–9.10)	0.042**^∗^**
	No	9 (47.37)	128 (79.14)	138 (75.82)	1 (reference)		1 (reference)	

Cigarette smoking history	Yes	2 (10.53)	13 (7.98)	15 (8.24)	1.36 (0.28–6.53)	0.70	—	—
	No	17 (89.47)	150 (92.02)	167 (91.76)	1 (reference)			

History of alcohol intake	Yes	7 (36.84)	34 (20.86)	41 (22.53)	2.21 (0.81–6.05)	0.12	1.85 (0.58–5.90)	0.131
	No	12 (63.16)	129 (79.14)	141 (77.43)	1 (reference)			

	Total (n/%)	19 (10.44)	163 (89.56)	182 (100)				

*Note:* NH, isoniazid; MDR, multidrug‐resistant; RIF, rifampicin; and TB–T2DM, tuberculosis with Type 2 diabetes mellitus.

Abbreviations: aOR, adjusted odds ratio; cOR, crude odds ratio; CI, confidence interval.

^∗^Statistically significant.

However, no significant associations were found for other tested variables, including sex, history of previous TB contact, cigarette smoking, and alcohol intake practices with an increased risk of developing MDR‐TB (*p* > 0.05) (Table 4).

## 4. Discussion

The emergence of DR in *M. tuberculosis* is caused by a low rate of chromosomal gene mutations, but this condition appears to be influenced by the internal environment of the host, the plasma concentration of anti‐TB drugs, insulin resistance, and co‐infection with other immune diseases such as HIV and diabetes [33]. The rising prevalence of TB–T2DM comorbidity and MDR‐TB presents a significant challenge to health systems in TB‐endemic countries. Our study identified a high proportion of TB–T2DM comorbidity (24.2%), which aligns with findings from India (29%) [11] and Mexico (35.5%) [16, 34]. This high prevalence is likely driven by hyperglycemia‐induced impairment of T‐lymphocyte function and reduced cellular immunity [10, 21]. Discrepancies between our findings and lower rates reported in Zambia (4.7%) [17], Southeastern Ethiopia (5.1%) [13], or Uganda (13.8%) [14] may be due to differences in DM screening methods, such as our use of HbA1c, which is more sensitive than fasting blood glucose alone in acute infection settings [13], or differences in sample size, study design, and study types [17].

In our study, the previous TB treatment (aOR: 2.51, 95% CI: 1.02–6.19, *p* = 0.046) and a family history of T2DM (aOR: 2.25, 95% CI: 1.10–4.60, *p* = 0.031) were strong predictors of TB–T2DM comorbidity. Our results were found comparable to the previous studies on the association of family history of diabetes [15, 34]. Notably, since our participants were newly diagnosed and treatment‐naïve at the time of screening, the hyperglycemia observed cannot be attributed to the hyperglycemic side effects of RIF or INH drugs [17]. Instead, this association suggests a shared genetic susceptibility or the role of chronic T2DM in predisposed individuals in increasing the likelihood of TB reactivation [34].

The results of this study did not find any significant association between the development of TB–DM comorbidity and patient characteristics such as age, sex, residency, previous contact with TB cases, window opening, alcohol drinking, and cigarette smoking practices (*p* > 0.05). This finding is similar to previous studies that presented no significant association with smoking [15], residency [13], alcohol drinking and smoking habits [16], and sex, age, and smoking [17]. However, contrary to our findings, previous studies identified a significant association between TB–T2DM comorbidity and patients with sex and age ≥ 50 years [13, 15, 16], previous contact with TB patients [34], and age ≥ 40 years [14]. The possible explanations for these associations might be due to a lack of physical activity during older age, which may be related to the significant increase in the prevalence of T2DM in this older age population and may reflect reduced cellular and humoral immunity as age advances, which increases the risk of developing TB–DM conditions and gestational DM during female’s previous pregnancy. Several studies have consistently reported that the discrepancies in these results could be due to the differences in the studied population, as they might have subjects with different sample sizes, age and gender distributions, diagnosis methods, and treatment durations [13–17].

Regarding genetic profiles, a total of 50 genetic mutations were distinguished within the *rpoB, katG,* and *inhA* promotor genes. Of these, 22 *rpoB*, 22 *katG,* and 6 *inhA* promoter mutations linked to RIF, high‐level, and low‐level INH resistance, respectively, were identified, with similar proportions between groups. Our variants’ distribution was in line with previous studies [10, 33, 35]. The *rpoB* S450L and *katG* S315T1 mutations were the most prevalent markers for RIF and INH resistance, respectively. Our finding is consistent with previous studies [11, 25, 33]. These mutations are typically associated with high‐level resistance and high biological fitness (low fitness cost), allowing for successful transmission within the community [23]. Most mutation patterns were similar between groups, and there were no significant differences observed in the number of genetic variants associated with resistance to INH, RIF, and MDR‐TB (*p* > 0.05), which is comparable with a previous study that showed no significant difference between the groups [33]. However, there was a significant presence of *rpoB* UN + *katG* S315T1 mutations conferring MDR‐TB in non‐diabetics versus TB–T2DM comorbidity (*p* = 0.04). The specific dual mutation in diabetics suggests that the internal metabolic environment of the diabetic host may influence the selection of specific resistance strains. Besides, the low occurrence of specific mutations in our study may affect the potential associations between mutation frequency and the presence of TB–T2DM comorbidity.

Our findings indicate that the presence of TB–T2DM comorbidity significantly increases the risk of developing MDR‐TB (aOR: 3.12, 95% CI: 1.02–9.10, *p* = 0.042), which is comparable to the previous studies that found a significant association [19, 34]. This risk may be impaired by altered drug pharmacokinetics in diabetic patients [21], potentially leading to subtherapeutic plasma concentrations of anti‐TB drugs and the subsequent selection of resistant mutants [19].

## 5. Conclusions

This study demonstrates that TB–T2DM co‐infection is a critical driver of DR in the Amhara Region of Ethiopia. The positive association observed between the presence of T2DM and MDR‐TB aligns with recent global studies suggesting that metabolic dysregulation may complicate TB treatment outcomes. While our cross‐sectional design prevents a causal conclusion, the increased odds of resistance in comorbid patients highlight the need for bidirectional T2DM screening for all TB patients, particularly those with a history of prior TB treatment or a family history of diabetes.

## 6. Limitations

This study has significant limitations. First, due to the cross‐sectional design, this study reports associations rather than causal relationships. Second, the analysis did not account for potential cofactors such as HIV status, nutritional status (BMI), and specific socioeconomic indicators, due to a lack of data at the time of the study. These variables are known to influence both TB susceptibility and treatment outcomes. Finally, the relatively small sample size of MDR‐TB isolates and the use of targeted LPAs rather than whole genome sequencing (WGS) suggest that the molecular mutation patterns should be interpreted as exploratory rather than definitive. Future studies should include these confounders in the multivariate model to provide a more comprehensive understanding of the association of TB–T2DM with the development of MDR‐TB. It is also essential to utilize WGS data and longitudinal designs to further elucidate the transmission dynamics and fitness cost of *M. tuberculosis* mutations in the context of chronic metabolic disorders.

**Nomenclature**
AOR: Adjusted odds ratio
CI: Confidence interval
COR: Crude odds ratio
DM: Diabetes mellitus
DR: Drug resistance
EDTA: Ethylenediamine‐tetra‐acetic acid
FBG: Fasting blood glucose
HbA1c: Glycosylated hemoglobin Type 1c
INH: Isoniazid
LJ: Löwenstein–Jensen
LMICs: Low‐ and middle‐income countries
LPA: Line probe assay
MDR‐TB: Multidrug resistant tuberculosis
NALC/NaOH: N‐Acetyl‐L‐cysteine/sodium hydroxide
NTM: Nontuberculosis mycobacteria
PTB: Pulmonary tuberculosis
RIF: Rifampin
T1DM: Type 1 diabetes mellitus
T2DM: Type 2 DM diabetes mellitus
TB: Tuberculosis
WGS: Whole genome sequencing
ZN: Ziehl–Neelsen

## Funding

The study was not funded.

## Ethics Statement

The study obtained ethical approval (Ref: VP/RTT/05/829) after reviewing the study proposal. Written informed consent was obtained from each subject who willingly participated in the study. The study was performed in accordance with the Declaration of Helsinki.

## Conflicts of Interest

The authors declare no conflicts of interest.

## Data Availability

The data that support the findings of this study are available from the corresponding author upon reasonable request.
